# Endodontic management of a maxillary first molar with unusual location of second mesio buccal orifice

**DOI:** 10.4103/0972-0707.71652

**Published:** 2010

**Authors:** R V S Chakradhar Raju, Naresh Sathe, Pradeep Kumar Morisetty, Chandrasekhar Veeramachaneni

**Affiliations:** Department of Conservative dentistry, Mamata Dental College, Khammam, Andhra Pradesh, India

**Keywords:** Anatomical variations, maxillary first molar, mesio-buccal root

## Abstract

Maxillary first molar can have a mesio-buccal-2 (MB2) orifice located palatally, but adjacent to the mesio buccal orifice. An awareness and understanding of this root canal morphology can contribute to the successful outcome of root canal treatment. This report discusses endodontic treatment of a maxillary first molar with unusual location of second mesio buccal orifice. Conventional diagnostic aids such as radiographs play an important role in assessment of complex root canal morphologies. These modalities, however, do not provide detailed information of the complexity as a result of their inherent limitations. This article discusses the variations in the orifice location and the use of latest adjuncts in successfully diagnosing and negotiating them.

## INTRODUCTION

A thorough knowledge of the root canal anatomy is a basic prerequisite for successful completion of the endodontic treatment.[1] Awareness and understanding of the presence of unusual external and internal root canal morphology contributes to the successful outcome of the root canal treatment. Maxillary molars are known to have an additional canal (MB2) in the mesio buccal root. The occurrence of second mesiobuccal canal is a common variation. Weine (2004) stated that frequent failure of endodontic treatment in maxillary first permanent molar teeth was likely due to the failure to locate and fill the second mesiobuccal canal.[2] Wolcott *et al*, have shown that failure to find and treat existing MB2 canal will decrease the long-term prognosis.[3,4]

Stropko conducted a study on 1096 maxillary first molars over an 8-year period and concluded that MB2 canals were found in 93% and 73.2% of first molars with and without the use of surgical operating microscopes.[5] Somma *et al*, studied the root canal morphology of 30 extracted human maxillary first molars with the aid of micro CT and concluded that the mesio buccal root canal anatomy was complex, with incidence of MB2 root canals, isthmuses, accessory canals, apical delta and loops.[6]

Complex root canal anatomies have been conventionally diagnosed by radiographs, which provide sufficient information to the clinician.[7] Although periapical and panoramic radiography produce acceptable details in the mesio-distal direction, the observation of details in the bucco-lingual dimension is inadequate.[8]

The present case report describes a case of a maxillary first molar with an unusual location of MB2 orifice and canal which is not yet reported in literature. An additional orifice was located adjacent to the palatal orifice which is an unusual occurrence.

## CASE REPORT

**Figure 1:**
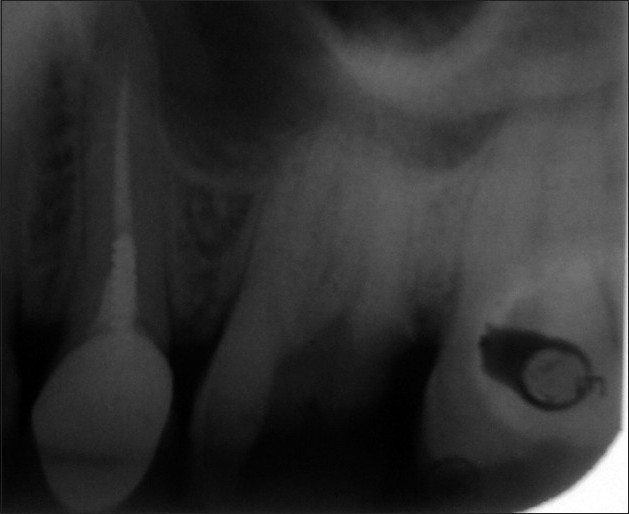
Pre operative IOPA showing decayed #14

**Figure 2:**
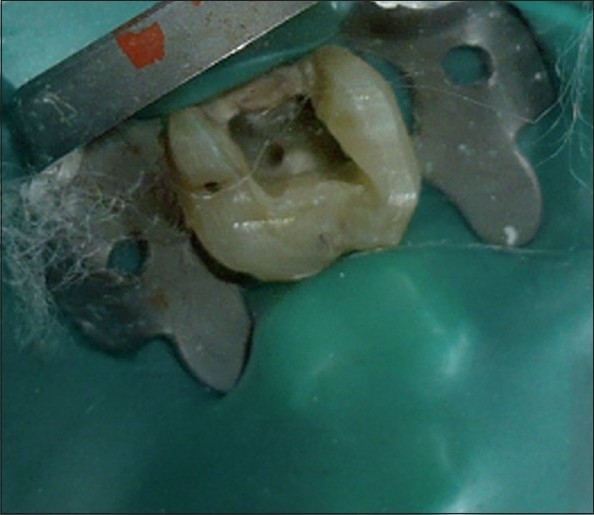
Intra oral photograph showing the access opening with four orifices with rubber dam in relation to #14

**Figure 3:**
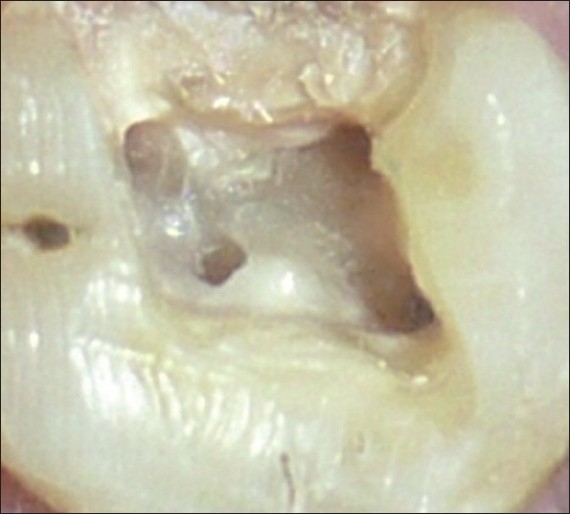
Intra oral photograph showing the access opening with four orifices without rubber dam in relation to #14

**Figure 4:**
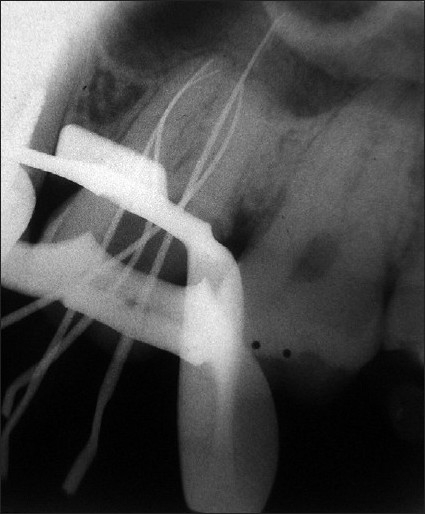
Intra oral radiograph showing working length in relation to # 14

A 45-year-old male patient reported with a chief complaint of continuous and radiating pain in relation to left maxillary first molar for several days. On clinical examination, the patient’s oral hygiene was found to be fair. Dental examination revealed a left maxillary first molar with a deep carious lesion and there was a full coverage restoration in relation to left maxillary second premolar. The patient also complained of episodes of sensitivity to hot and cold in the involved tooth. Clinical diagnosis was irreversible pulpitis. A preoperative radiograph was obtained [Figure 1]. After detailed clinical and radiographic examination, the left maxillary first molar was prepared for nonsurgical endodontic therapy. After administration of local anesthesia, tooth was isolated with a rubber dam and a conventional endodontic access opening was made [Figure 2]. After removing pulp tissue located in the chamber, four orifices were observed – palatal, mesiobuccal and distobuccal located in regular locations and an extra orifice was located very close to the palatal orifice and was presumed to be a second palatal orifice. The conventional triangular access was modified to a trapezoidal shape to improve access to the additional canal [Figure 3]. The working length of each canal was estimated by means of an electronic apex locator (Root ZX; Morita, Tokyo, Japan) and then confirmed by a radiograph [Figure 4] and then access was closed with a temporary restoration.

There were no variations like second palatal root/ canal observed in the pre-operative radiograph and working length radiograph suggested that the instrument in the extra canal was an MB2, but at an unusual location i.e., adjacent to palatal orifice.

**Figure 5:**
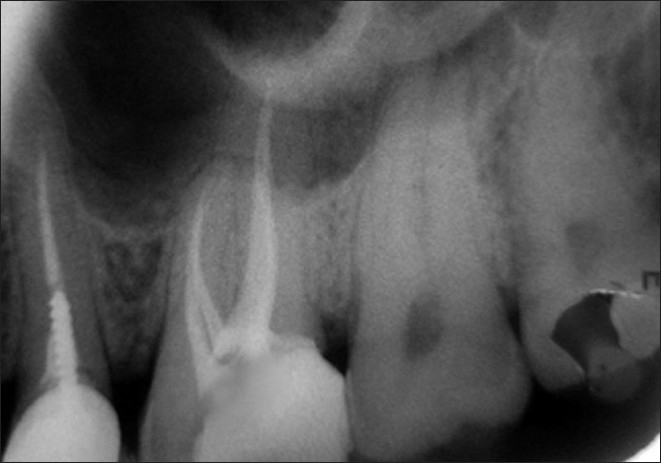
Intra oral radiograph showing obturation of the four canals in relation to #14

At the next visit, the canals were initially instrumented with #15 nickel titanium files (Dentsply Maillefer) under irrigation with 5% sodium hypochlorite and 17% EDTA. Coronal flaring was carried out by using gates glidden burs (numbers 3 and 2; Dentsply Maillefer). Cleaning and shaping of the canals was done by using hand nickel titanium Protaper file system with a crown-down technique similar to that described by Saunders and Saunders.[9] The canals were obturated with AH plus resin sealer (Dentsply Maillefer, Ballaigues, Switzerland) and gutta-percha points using lateral condensation technique. The access cavity was then restored with posterior composite filling (P60; 3M Dental Products, St. Paul, MN) [Figure 5].

## DISCUSSION

Majority of endodontic literature describe the maxillary first molar as having three roots and four root canals, with two canals in mesiobuccal root. In most of the teeth, the location of MB2 orifice opening is usually found mesial to an imaginary line between the MB1 and palatal orifices, and at about 2 to 3 mm from the MB1 orifice. This report highlights the unusual presence of MB2 canal orifice in maxillary first molar adjacent to the palatal orifice which was not reported in literature till date.

Tachibana concluded that applicability of computed tomography (CT) for endodontics allowed the observation of the morphology of the root canals, the roots, and the appearance of the tooth in every direction.[10] Gurmeet Singh *et al*, have used SCT for the confirmatory diagnosis of morphological aberrations in the root canal anatomy.[11] In the present case, working length radiograph revealed that the orifice was that of MB2 canal.

Of all the canals in the maxillary first molar, the MB2 can be the most difficult to find and negotiate in a clinical situation. Instrumentation of this tooth, especially with respect to the mesiobuccal root, can be complicated. Failure to detect and treat the second MB2 canal system will result in a decreased long-term prognosis. Stropko observed that by scheduling adequate clinical time, by using the recent magnification and detection instrumentation aids and by having thorough knowledge of how and where to search for MB2, the rate of location can approach 93% in maxillary first molars.[12]

## CONCLUSION

Additional canal such as MB2 in maxillary molar is a frequently encountered clinical situation. Usually, this additional canal is located adjacent to MB1 but in this instance, it is found to be adjacent to palatal orifice. Such aberrant location and confirmation were possible with the use of intra oral periapical radiograph. The finality in locating the canal especially in challenging situations buttresses the need for use of non-invasive and advanced gadgets such as CT.
